# Intra-tracheal administration of a naked plasmid expressing stromal derived factor-1 improves lung structure in rodents with experimental bronchopulmonary dysplasia

**DOI:** 10.1186/s12931-019-1224-6

**Published:** 2019-11-12

**Authors:** Kasonya Guerra, Carleene Bryan, Frederick Dapaah-Siakwan, Ibrahim Sammour, Shelly Drummond, Ronald Zambrano, Pingping Chen, Jian Huang, Mayank Sharma, Sebastian Shrager, Merline Benny, Shu Wu, Karen C. Young

**Affiliations:** 1 Department of Pediatrics, University of Miami Miller School of Medicine, 1580 NW 10th Avenue RM-344, Miami, FL 33136 USA; 2 Batchelor Children’s Research Institute, University of Miami Miller School of Medicine, 1580 NW 10th Avenue RM-344, Miami, FL 33136 USA; 3 The Interdisciplinary Stem Cell Institute, University of Miami Miller School of Medicine, 1580 NW 10th Avenue RM-344, Miami, FL 33136 USA

**Keywords:** Bronchopulmonary dysplasia, Hyperoxia, Angiogenesis, Stromal derived factor-1

## Abstract

**Background:**

Bronchopulmonary dysplasia (BPD) is characterized by alveolar simplification and disordered angiogenesis. Stromal derived factor-1 (SDF-1) is a chemokine which modulates cell migration, proliferation, and angiogenesis. Here we tested the hypothesis that intra-tracheal (IT) administration of a naked plasmid DNA expressing SDF-1 would attenuate neonatal hyperoxia-induced lung injury in an experimental model of BPD, by promoting angiogenesis.

**Design/methods:**

Newborn Sprague-Dawley rat pups (*n* = 18–20/group) exposed to room air (RA) or hyperoxia (85% O2) from postnatal day (P) 1 to 14 were randomly assigned to receive IT a naked plasmid expressing SDF-1, JVS-100 (Juventas Therapeutics, Cleveland, Ohio) or placebo (PL) on P3. Lung alveolarization, angiogenesis, inflammation, vascular remodeling and pulmonary hypertension (PH) were assessed on P14. PH was determined by measuring right ventricular systolic pressure (RVSP) and the weight ratio of the right to left ventricle + septum (RV/LV + S). Capillary tube formation in SDF-1 treated hyperoxia-exposed human pulmonary microvascular endothelial cells (HPMEC) was determined by matrigel assay. Data is expressed as mean ± SD and analyzed by two-way ANOVA.

**Results:**

Exposure of neonatal pups to 14 days of hyperoxia decreased lung SDF-1 gene expression. Moreover, whilst hyperoxia exposure inhibited capillary tube formation in HPMEC, SDF-1 treatment increased tube length and branching in HPMEC. PL-treated hyperoxia-exposed pups had decreased alveolarization and lung vascular density. This was accompanied by an increase in RVSP, RV/LV + S, pulmonary vascular remodeling and inflammation. In contrast, IT JVS-100 improved lung structure, reduced inflammation, PH and vascular remodeling.

**Conclusions:**

Intratracheal administration of a naked plasmid expressing SDF-1 improves alveolar and vascular structure in an experimental model of BPD. These findings suggest that therapies which modulate lung SDF-1 expression may have beneficial effects in preterm infants with BPD.

## Background

Bronchopulmonary dysplasia (BPD) is the leading cause of chronic lung disease in infancy. Since its first description in 1967 [1], the incidence of BPD has remained high as more extremely low birth weight premature infants survive [2–4] and few therapeutic interventions have reduced the disease burden. BPD survivors have an increased risk of pulmonary hypertension (PH), growth failure, neuro-developmental delay and other long-term sequelae causing a significant impact on families and health care systems [5, 6]. Inflammation and altered angiogenic signaling play a key role in the development of BPD [7] and strategies which modulate these pathways may reduce BPD incidence.

Stromal derived factor-1 (SDF-1), also called chemokine ligand 12 (CXCL12), is a small pleiotropic molecule belonging to the CXC chemokine family. It is encoded by the CXCL12 gene on chromosome 10 and is constitutively expressed by many tissues and cell types [8]. Its main receptors are chemokine receptor 4 (CXCR4) and chemokine receptor 7 (CXCR7). Within the lung, SDF-1 is expressed by several cell types including the epithelium, while its receptors CXCR4 and CXCR7 are expressed on vascular endothelial cells. In addition, CXCR7 is also expressed on the epithelium [9–11]. SDF-1 signaling culminates in a multitude of biological functions including modulation of chemotaxis, proliferation, apoptosis, survival, and differentiation [12, 13].

SDF-1 plays an important role in organogenesis and early development. SDF-1 knockout mice exhibit embryonic lethality with survivors having cardiac defects, impairment in B-cell lymphopoiesis and bone marrow myelopoiesis during embryonic development [14]. CXCR4 deficient mice die perinatally and have similar defects in hematopoietic and cerebellar development [15]. Additionally, CXCR7 knockout mice die postnatally with survivors having cardiac abnormalities [10]. In the lung, SDF-1 conditional knockout neonatal mice have abnormal lung structure with an increase in alveolar spaces, whilst adult mice exhibit emphysematous changes in lung morphology [16].

The role of SDF-1 in organ injury and repair has been controversial. Indeed, while several reports demonstrate that SDF-1 and/or its receptors promote repair of the injured kidney, liver, limb, brain and retina [17–23] by mobilizing endothelial progenitor cells to injury sites [22, 24, 25] or upregulating vascular endothelial growth factor (VEGF) and other angiogenic factors [26], in adult lung disease models, SDF-1/CXCR4 signaling potentiates lung inflammation and CXCR4 antagonism attenuates lung inflammation and injury [27, 28]. Xu and colleagues [27] demonstrated that SDF-1 levels and CXCR4^pos^ cells are increased in patients with idiopathic pulmonary fibrosis and antagonism of CXCR4, attenuates bleomycin-induced lung fibrosis. Conversely, SDF-1 inhibition impairs alveolar epithelial cell spreading and delays resolution of permeability after lung injury [29] and agonism of the SDF-1 receptor, CXCR7, prevents epithelial damage, promotes alveolar repair and reduces bleomycin-induced pulmonary fibrosis [30]. There is also new evidence demonstrating that platelet-derived SDF-1 plays a crucial role in lung regeneration after pneumonectomy [31]. While these discrepancies maybe secondary to differences in the models of injury as well as the relative concentration and structural conformation of SDF-1, more recent reports show differential signalling of SDF-1 through its receptors during the acute and chronic stages of injury [30].

Whether SDF-1 modulates neonatal hyperoxia-induced lung injury was heretofore unknown. SDF-1 protein expression is however unchanged in neonatal rodents exposed to hyperoxia suggesting that suboptimal SDF-1 lung levels may blunt the intrinsic reparative response following neonatal hyperoxia exposure [32]. In the present study, we hypothesized that augmentation of the intrinsic SDF-1 response in neonatal rodents exposed to hyperoxia, by intra-tracheally (IT) administering a naked plasmid DNA expressing SDF-1 (JVS-100), would attenuate lung injury by promoting angiogenesis. JVS-100 (Juventas Therapeutics, Cleveland, Ohio) is a non-viral gene therapy engineered to express SDF-1. In a porcine model, JVS-100 was effectively delivered to the myocardium with efficient gene uptake and significant gene expression [33]. The safety and potential efficacy of JVS-100 in patients with ischemic cardiomyopathy have also been shown in recent phase 1 and 2 clinical trials [34, 35].We demonstrate increased capillary tube formation following SDF-1 treatment of hyperoxia-exposed pulmonary microvascular endothelial cells (HPMECs). In a neonatal rodent model of BPD, we demonstrate that IT JVS-100 improves angiogenesis, attenuates PH and decreases inflammation. These findings suggest a potential therapeutic role of SDF-1 in the repair of the injured preterm lung.

## Materials and methods

### Animals

Pregnant Sprague Dawley rats were purchased from Charles River Laboratories (Wilmington, MA). Animals were treated according to National Institute of Health (NIH) guidelines for the use and care of laboratory animals following approval of the study protocol by the University of Miami Animal Care and Use Committee.

### Experimental design

Sprague Dawley pups assigned to normoxia (RA) or hyperoxia (85% O_2_) from postnatal day (P)1 to P14 were randomly assigned to receive intratracheal (IT) injections of a plasmid expressing SDF-1 (JVS-100;100 μg/50 μl) or phosphate buffered saline as placebo (PL) on P3. This dose was chosen following pilot studies in our laboratory. Following anesthesia with isoflurane, the trachea was exposed through a small incision in the midline of the neck, and IT JVS-100 or PL were delivered by tracheal puncture with a 30-gauge needle. The incision was closed with Vetbond™ tissue adhesive (3 M, St. Paul, MN) and the pups were allowed to recover within a warmed plastic chamber**.** Oxygen exposure was achieved in a Plexiglas chamber by a flow-through system and the oxygen level inside the chamber was monitored daily with a Maxtec oxygen analyzer (Model OM25-RME; Maxtec, Salt Lake City, Utah). Mothers were rotated every 48 h between the hyperoxia and normoxia chambers to prevent damage to their lung. Litter size was adjusted to 10–12 pups to control for the effect of litter size on nutrition and growth. Hemodynamic measurements, lung morphometric and molecular studies were performed on P14.

In a subgroup of animals in order to determine whether IT administration of a plasmid would be an efficient technique to deliver SDF-1 to the lungs, pups received IT injections of a naked plasmid encoding luciferase (pLuc-100 μg/50 μl) on P3. On P5 and 14, luciferin (150 mg/kg) was injected intraperitoneally (IP). The pups were anesthetized using 4 ppm of inhaled isoflurane via the VetFlo anesthesia system (Kent Scientific Corp, Torrigton,CT) and real-time images using Xenogen IVIS Spectrum (PerkinElmer, Inc. Waltham, MA) were obtained. Following imaging, pups were euthanized and the lungs removed for immunostaining and molecular studies.

### Lung morphometric analysis

Pulmonary morphometry was performed as previously described [36]. Briefly, the trachea and pulmonary artery were perfused and fixed overnight in 4% paraformaldehyde and embedded in paraffin. Serial sections 5 μm thick were taken from the upper and lower lobes and stained with hematoxylin and eosin. Images from randomly selected, non-overlapping parenchymal fields were acquired from lung sections of each animal using an Olympus Qcolor 3 color camera interfaced with a light microscope (Model Leica DMI 4000B). Alveolarization was determined by calculating the radial alveolar count and septal thickness. The number of alveoli transected by a perpendicular line drawn from the center of a respiratory bronchiole to the nearest septal division or pleural margin was used to determine the radial alveolar count. Septal thickness was assessed on hematoxylin and eosin stained lung sections by averaging 100 measurements per 10 representative fields at X 400 magnification.

### Pulmonary vascular density

Vascular density was evaluated as previously described [37]. Lung sections were de-paraffinized, rehydrated, and stained with polyclonal rabbit anti-human Von Willebrand Factor (vWF,1:200; Dako Corp, Carpintaria, CA). The number of vessels (20–50 μm diameters) per high power field (HPF) was quantified in 5 randomly selected, non-overlapping, parenchymal fields from lung sections of each animal.

### Pulmonary vascular remodeling

Pulmonary vascular remodeling was evaluated as previously described [36]. Briefly, lung sections were stained with polyclonal rabbit anti-human vWF (Dako), mouse anti α-smooth muscle actin (SMA,1:500; Sigma, St Louis, MO) and 4′,6-diamidino-2-phenylindole (DAPI, Vector Biolabs, Burlingame, CA). The percentage of peripheral pulmonary vessels (< 50 μm in diameter) stained with α-SMA > 50% of the circumference was determined from ten random images on each lung section and all analyses were performed by a blinded observer.

### Hemodynamic studies

Pups were anesthetized and right ventricular systolic pressure (RVSP) evaluated as previously described [36]. Briefly, a thoracotomy was performed and a 22 gauge needle connected to a pressure transducer was inserted into the right ventricle. RVSP was measured and recorded on a Gould polygraph (Model TA-400, Gould instruments, Cleveland,OH). Right ventricular hypertrophy (RVH) was determined by measuring the weight ratio of the right ventricle (RV) to the left ventricle (LV) and septum (S).

### Lung inflammation

Broncho-alveolar lavage fluid (BAL) analysis was performed as previously described [38]. Briefly, a 20 gauge angiocatheter was inserted into the trachea and secured in place with a 4.0 silk suture. The lungs were lavaged by infusing and then aspirating four aliquots of normal saline (0.5 ml each) into the lungs. The BAL obtained was then centrifuged for 5 min. The cells were washed with normal saline and quantified using a hemocytometer. Cells were suspended in 1000 μl of normal saline and affixed to slides using an Eppendorf centrifuge. BAL cell counts were performed on the cytospin preparations after Giemsa staining. Lung inflammation was also assessed by immunostaining for MAC-3,a macrophage-specific marker, using a monoclonal rat antibody obtained from BD Biosciences (1:20,San Jose, CA). The number of MAC-3^pos^ cells in the alveolar air spaces was counted from 10 random images taken with the 20X objective on each slide.

### Real time RT-PCR

Total RNA was isolated from lung tissue stabilized in RNAlater using RNeasy Midi Kit (Qiagen, Inc. Valencia, CA) as per manufacturer instructions. Tissues were disrupted and homogenized using a rotor-stator-homogenizer (Ultra-Turrax T8, IKA Works, Wilmington, NC). Samples were centrifuged for 15 min at 12,000×g at 4 °C. The upper aqueous phase was transferred to a new collection tube, and an equal volume of 70% ethanol was added and vortexed. Following step centrifugation at 10,000×g, RNA was eluted using 30~50 μl RNase-free water. RNA purity and concentration were determined by NanoDrop 1000 Spectrophotometer (Thermo Fisher Scientific, Waltham, MA). Total RNA (2 μg) was reverse transcribed using a first-strand cDNA synthesis kit according to manufacturer’s protocol (Superscript VI VILO Master Mix with ezDNase Ensyme, Thermo Fisher Scientific). This kit contains ezDNase enzyme which is a double-strand specific thermolabile DNase that is used to remove gDNA contamination from template RNA prior to the RT reaction. Real time RT-PCR using TaqMan™ Fast Advanced Master Mix (Applied Biosystems, Foster City, CA) was performed on an ABI Fast 7500 system (Applied Biosystems) using a standard cycling protocol. Primers for rat SDF-1 (Rn00573260), 18S (Rn4332641) and GAPDH (Rn99999916) were pre-developed by Applied Biosystems. The mRNA expression levels of target genes were normalized to 18S and GAPDH. Since either normalization yielded similar results, data are reported with normalization to 18S.

### Western blot analysis

The protein expression of Interleukin-1β (IL-1β), Interleukin-10 (IL-10), vascular endothelial growth factor receptor-2 (VEGFR-2), and SDF-1 in lung homogenates was determined by Western Blot analysis. The polyclonal antibodies for VEGFR2 (1:200) and IL-10 (1:2000) were obtained from Abcam (Cambridge, MA). The polyclonal antibody for SDF-1 (1:50) and mouse monoclonal antibody for IL-1β (1:1000) were obtained from Cell Signaling Technology (Danvers, MA). Total protein was extracted from frozen lung tissues with a RIPA buffer according to the manufacturer’s protocol (Santa Cruz, Dallas, TX). Protein concentration was measured by BCA protein assay using a commercial kit from Pierce Biotechnology Inc. (Rockford, IL). Lung lysate (50 μg/sample) were fractionated by SDS-PAGE on 4–20% mini-protean Tris-Glycine extended precast protein gel (Bio-Rad, Hercules, CA) and transferred to nitrocellulose membranes (Amersham, Piscataway, NJ). Immunodetection was performed by incubating the membranes with the primary antibodies diluted in blocking buffer overnight at 4 °C and then for 1 h at room temperature with horseradish peroxidase-cojugated secondary antibodies. Antibody bound protein was detected using ECL chemiluminescence methodology (Amersham). Band intensity was quantified with Quantity One software (Bio-Rad), with β-Actin acting as the normalization protein (1:10,000; Sigma-Aldrich, St. Louis, MO).

### Immunohistochemistry and immunofluorescence

Serial five micrometer (μm) paraffin-embedded lung sections were dewaxed and rehydrated in descending grades of alcohol. Following antigen retrieval and blocking of non-specific binding sites with a protein blocker, the lung sections were incubated overnight at 4 °C with the appropriate primary antibodies. After washing, the sections were incubated with the appropriate secondary antibodies at room temperature. Sections were evaluated under a fluorescent microscope (Leica DMI 6000, Mannheim, Germany).

### Matrigel assay

The effect of SDF-1 on capillary tube formation was determined by matrigel assay as previously described [39]. HPMECs (Lonza, Allendale, NJ) were cultured to passage 3 to 6, plated in 100 mm dishes and serum starved for 48 h. Serum-starved HPMECs were treated with varying doses of recombinant SDF-1 (10–100 ng/ml) and cultured in normoxic (RA, 5% CO_2_) or hyperoxic (95% O_2_, 5% CO_2)_ conditions for 72 h. Capillary tube formation was assessed on growth factor reduced matrigel-coated wells (BD Biosciences, San Diego, CA). Bright field images were collected at 5 and 20 h. All experiments were done in triplicate and tube formation was quantified by measuring the number and length of capillary-like structures in at least three HPFs per well.

### Statistical analysis

Results, reported as mean ± standard deviation (SD), were analyzed by two-way ANOVA with post-hoc Holm-Sidak test using SigmaStat software. *P* values less than 0.05 were considered statistically significant.

## Results

### Effect of hyperoxia on lung SDF-1 expression

**Fig. 1:**
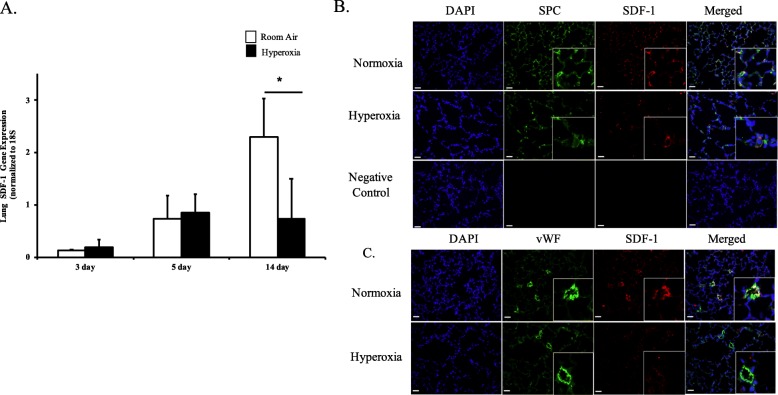
The effect of hyperoxia on lung SDF-1 expression. **a** Decreased lung SDF-1 gene expresion in newborn pups exposed to 14 d of hyperoxia (*P* < 0.05; *Normoxia vs hyperoxia; *N* = 4–5 animals/group). **b** Lung sections obtained from 14 day old normoxic and hyperoxic pups stained with SDF-1 (red) and SPC (green) antibodies. SDF-1^pos^SPC^pos^ cells (yellow) were more abundant in normoxic pups. **c** Lung sections stained with SDF-1 (red) and vWF (green) antibodies. SDF-1^pos^vWF^pos^ cells (yellow) were more abundant in normoxic pups. Scale bar is 50 μm and original magnification is X200

We first evaluated SDF-1 gene expression in lung homogenates of neonatal pups exposed to 3, 5 and 14 days of hyperoxia. Exposure of neonatal pups to 3 or 5 days of hyperoxia did not alter lung SDF-1 gene expression, Fig. 1a. However, following 14 days of hyperoxia exposure, there was a significant decrease in lung SDF-1 gene expression (RA vs hyperoxia; *P* = 0.03; *N* = 4–5/group), Fig. 1a. Double immunofluorescence staining of lung sections with SDF-1 and surfactant protein C or vWF antibodies revealed that SDF-1 is expressed in both lung epithelial and endothelial cells, Fig. 1b and c.

### Effective pulmonary delivery of IT JVS-100

**Fig. 2:**
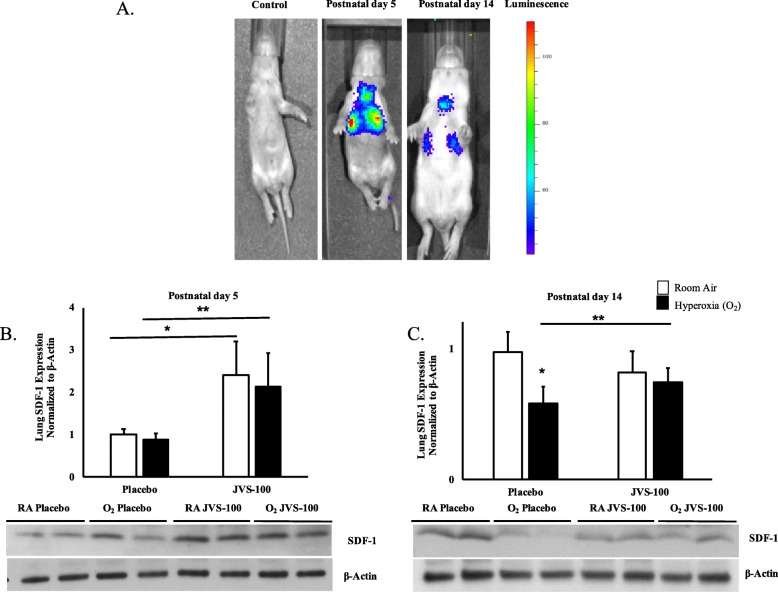
The effective pulmonary delivery of JVS-100. **a** Representative images of luciferase activity in the lungs of P5 and P14 rats who received PBS (control) and pLuc. **b** Increased SDF-1 protein expression in lung homogenates of P5 rats and (**c**) P14 rats who received IT JVS-100. SDF-1 expression was normalized to β-Actin. RA is room air and O_2_ is hyperoxia. *P* < 0.05; * RA-PL vs RA-JVS-100 or hyperoxia-PL; ** hyperoxia-PL vs hyperoxia-JVS-100; *N* = 4–5 animals /group. A representative western blot is shown in the lower panel.

In order to ascertain whether IT administration of a naked plasmid would be an efficient technique to deliver SDF-1 to the lungs, Sprague Dawley pups were given a plasmid expressing luciferase on P3. Significant luciferase activity was detected in the lung on P5, Fig. 2a. While there was still residual activity detected on P14, this was decreased, Fig. 2a. Western blot analysis of P5 and P14 lung homogenates confirmed increased SDF-1 protein expression in oxygen exposed rats who received JVS-100, Fig. 2b and c.

### JVS-100 improves lung alveolarization in experimental BPD

**Fig. 3:**
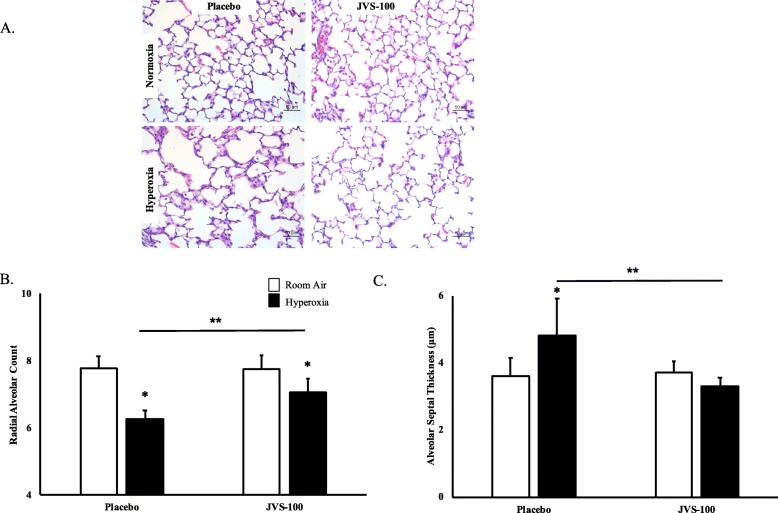
JVS-100 improves lung alveolarization. **a** Haematoxylin and eosin stained lung sections obtained from P14 rats demonstrating improved alveolar structure in hyperoxia-exposed pups treated with IT JVS-100. Original magnification X100. Scale bars are 100 μm. **b** Morphometric analyses revealed an increase in radial alveolar count and (**c**) reduced alveolar septal thickness in hyperoxia-exposed pups treated with IT JVS-100 (*P* < 0.05; * RA-PL vs hyperoxia-PL or hyperoxia-JVS-100; ** hyperoxia-PL vs hyperoxia-JVS-100; *N* = 14–19 animals/group)

Hyperoxia-exposed placebo-treated (Hyperoxia-PL) pups had decreased alveolarization as evidenced by alveolar simplification, Fig. 3a. Radial alveolar count was utilized as a morphometric measure of alveolarization. Whereas hyperoxia-PL pups had a decrease in radial alveolar count (8 ± 0.3 vs 6 ± 0.3; RA-PL vs hyperoxia-PL; *P* < 0.05; *N* = 14–19 animals/group), Fig. 3b, administration of IT JVS-100 increased radial alveolar count in the hyperoxia-exposed pups (6 ± 0.3 vs 7 ± 0.4; hyperoxia-PL vs hyperoxia-JVS-100; *P* < 0.05; *N* = 14–19 animals/group), Fig. 3b. Similarly, whereas hyperoxia-PL treated pups had an increase in alveolar septal thickness, this was reduced in JVS-100 treated pups, Fig. 3c.

### JVS-100 improves angiogenesis in experimental BPD

**Fig. 4:**
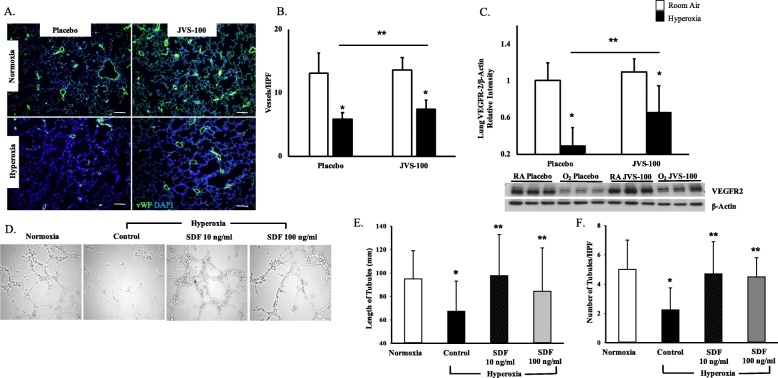
JVS-100 improves lung angiogenesis. **a** Lung sections stained with Von Willebrand Factor (green) demonstrating improved vascular density in hyperoxia-exposed pups treated with IT JVS-100. Original Magnification X100. Scale bars are 100 μm. **b** Hyperoxia exposure decreased vascular density but the administration of IT JVS-100 was associated with improved angiogenesis (*P* < 0.05; * RA-PL vs hyperoxia-PL or hyperoxia-JVS-100; **hyperoxia-PL vs hyperoxia-JVS-100; *N* = 10 animals/group). **c** Increase in lung VEGFR-2 expression in hyperoxia-exposed JVS-100 treated pups. A representative Western Blot is shown in the lower panel with VEGFR-2 expression normalized to β-Actin (*P* < 0.05; * RA-PL vs hyperoxia-PL or hyperoxia-JVS-100; **hyperoxia-PL vs hyperoxia-JVS-100; *N* = 6 animals/group) (**d**) Increased tubule formation 20 h after plating recombinant SDF-1 treated hyperoxia-exposed HPMEC on matrigel. Original Magnification X 100. Scale bar is 50 μm. Increase in the length (**e**) and number of cord-like structures (**f**) per high power field (HPF) in hyperoxia-exposed recombinant SDF-1 treated HPMECs (*P* < 0.05; *RA vs hyperoxia-control; ** hyperoxia-control vs hyperoxia recombinant SDF-10 ng/ml or hyperoxia recombinant SDF-100 ng/ml; all experiments were performed in triplicate). RA is room air and O_2_ is hyperoxia

SDF-1 plays a crucial role in angiogenesis [26]. Thus, we next questioned whether IT JVS-100 would improve angiogenesis in neonatal rats exposed to hyperoxia. Exposure of neonatal pups to hyperoxia reduced vascular density, Fig. 4a and b, as evidenced by decreased number of vessels per HPF (13 ± 3 vs 5.8 ± 0.9 vessels/HPF; RA-PL vs hyperoxia-PL; *P* < 0.05; *N* = 10 animals/group). However, IT administration of JVS-100 modestly improved lung angiogenesis (5.8 ± 0.9 vs. 7.4 ± 1.4 vessels/HPF; hyperoxia-PL vs hyperoxia-JVS-100; *P* < 0.05; *N* = 10 animals/group), Fig. 4a and b. This was accompanied by a significant increase in lung VEGFR-2 expression in the hyperoxic JVS-100 treated pups (hyperoxia-PL vs hyperoxia-JVS-100; *P* < 0.05; *N* = 6 animals/group), Fig. 4c. There was no difference in VEGF expression between the hyperoxia groups. In order to confirm the direct pro-angiogenic effects of SDF-1, hyperoxia-exposed HPMECs were treated with varying doses of recombinant SDF-1 (10 or 100 ng/ml) and matrigel assay performed. Hyperoxia-exposed HPMECs had significantly decreased length and number of capillary-like structures. Treatment with recombinant SDF-1 (10 or 100 ng/ml) promoted angiogenesis in hyperoxia-exposed HPMECs as evidenced by increased length and number of capillary-like structures (hyperoxia control vs hyperoxia SDF-10 ng/ml or hyperoxia SDF-100 ng/ml; *P* < 0.05; all experiments performed in triplicate), Fig. 4d-f. There was no significant effect in the normoxia exposed cells.

### JVS-100 attenuates pulmonary hypertension

**Fig. 5:**
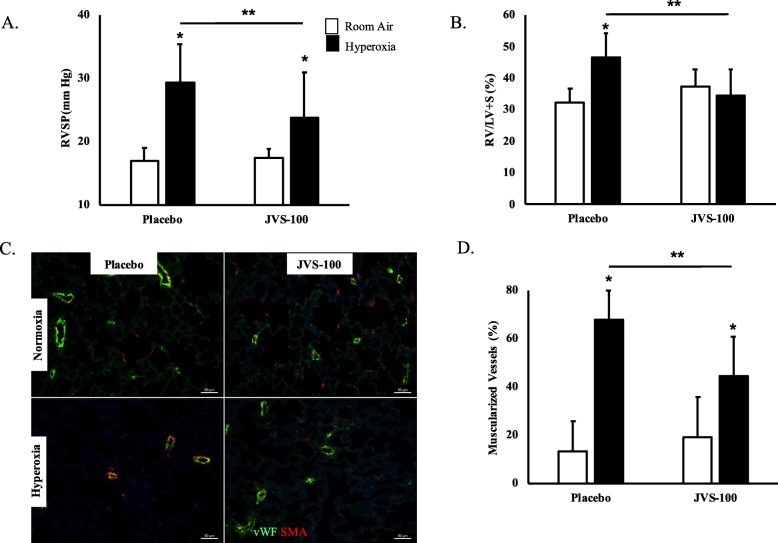
Effects of JVS-100 on pulmonary hypertension and vascular remodeling in experimental BPD. IT JVS-100 significantly decreased (**a**) right ventricular systolic pressure (RVSP) and (**b**) RV/LV + S (weight ratio of right ventricle to left ventricle and septum) in hyperoxia-exposed animals (**c**) Lung sections stained with α-smooth muscle actin (red) demonstrating improved vascular remodeling in hyperoxia-exposed pups treated with IT JVS-100. Magnification X 200. Scale bars are 50 μm. (**d**) Reduced muscularized vessels in the lungs of hyperoxia-exposed animals after IT JVS-100. (*P* < 0.05; * RA-PL vs hyperoxia-PL or hyperoxia-JVS-100; ** hyperoxia-PL vs hyperoxia-JVS-100; *N* = 19–20 animals/group)

Neonatal pups exposed to hyperoxia developed evidence of PH (RVSP: 17 ± 2 vs 29 ± 6 mmHg; RA-PL vs hyperoxia-PL; *P* < 0.05; *N* = 19–20 animals/group and RV/LV + S (0.32 ± 0.05 vs 0.46 ± 0.08 mmHg; RA-PL vs hyperoxia-PL; *P* < 0.05; *N* = 19–20 animals/group), Fig. 5a and b. Both RVSP and RV/LV + S were reduced after the IT administration of JVS-100 in the hyperoxia-exposed neonatal pups (RVSP: 29 ± 6 vs 24 ± 7 mmHg; hyperoxia-PL vs hyperoxia-JVS-100; *P* < 0.05; *N* = 19–20/group and RV/LV + S: 0.46 ± 0.08 vs 0.34 ± 0.08 mmHg; hyperoxia-PL vs hyperoxia-JVS-100; *P* < 0.05; *N* = 19–20/group), Fig. 5a and b. Together these findings suggest that IT JVS-100 improves PH, a significant component of severe BPD.

Vascular remodeling evidenced by increased muscularized blood vessels is a prominent feature of BPD complicated by PH. Exposure of placebo treated animals to hyperoxia was associated with an increase in the percentage of muscularized blood vessels (13 ± 12 vs 67 ± 12%; RA-PL vs hyperoxia-PL; *P* < 0.05; *N* = 6 animals/ group), Fig. 5c and d. In contrast, administration of IT JVS-100 improved hyperoxia-induced pulmonary vascular remodeling in experimental BPD (67 ± 12 vs 44 ± 16%; hyperoxia-PL vs hyperoxia-JVS-100; *P* < 0.05; *N* = 10/group), Fig. 5c and d.

### JVS-100 attenuates lung inflammation in experimental BPD

**Fig. 6:**
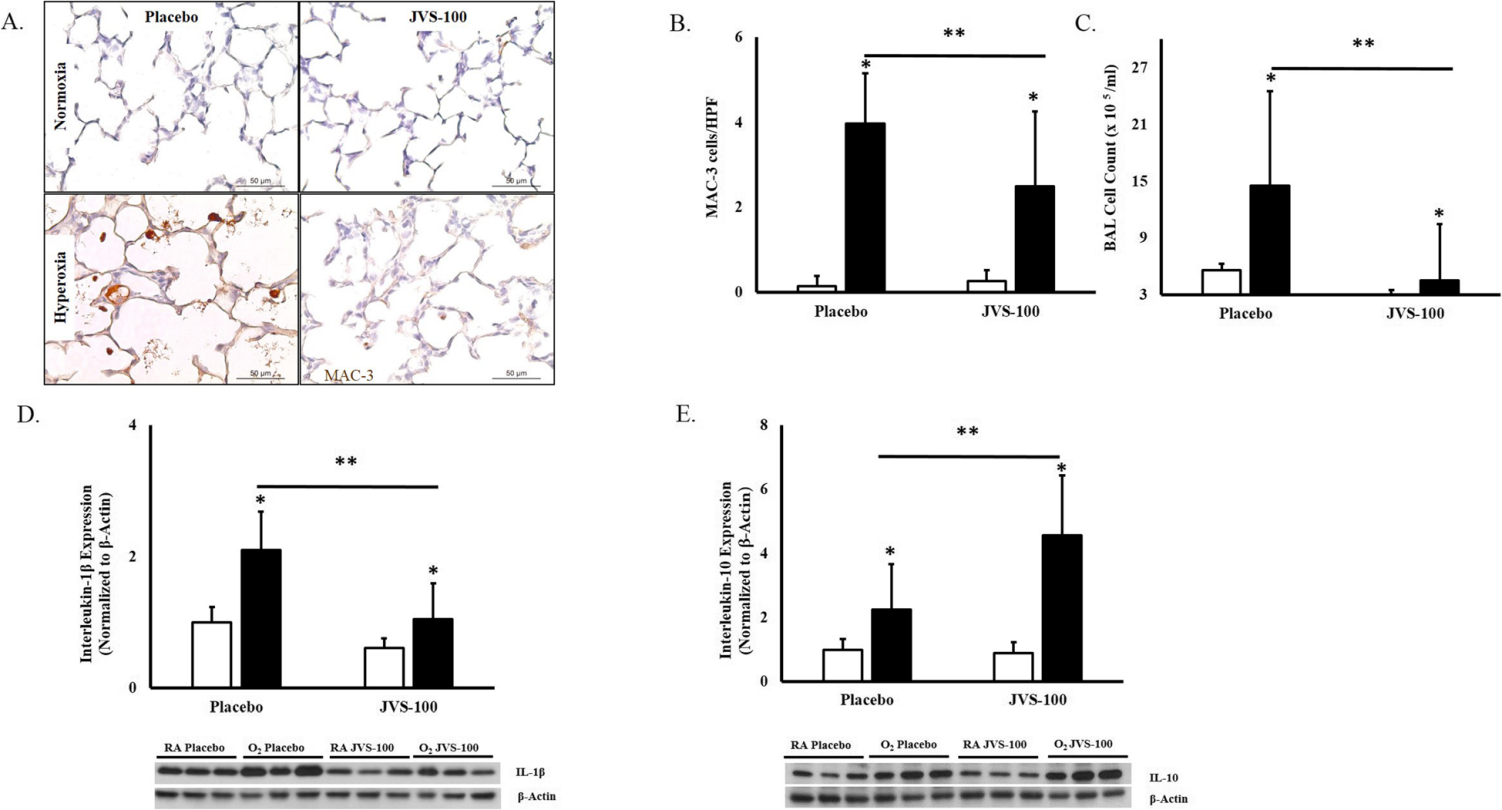
Anti-inflammatory effects of JVS-100 in experimental BPD. **a** Lung sections stained with MAC-3 (brown) showing increased MAC-3 positive cells/HPF in hyperoxia-exposed pups. Original magnification X 200. Scale bars are 50 μm. **b** IT JVS-100 significantly reduced MAC-3^pos^ cells/HPF and (**c**) BAL cell count in hyperoxia-exposed pups. **d** Decrease in the pro-inflammatory cytokine IL-1β and (**e**) increase in the anti-inflammatory cytokine, IL-10 in the hyperoxia-exposed JVS-100 treated pups (*P* < 0.05; * RA-PL vs hyperoxia-PL or hyperoxia-JVS-100, **hyperoxia-PL vs hyperoxia-JVS-100; *N* = 6 animals/group). A representative Western blot is shown in the lower panel normalized to β-Actin. RA is room air and O_2_ is hyperoxia

Lung inflammation is a key feature of BPD. Hyperoxia-PL treated pups had an increase in MAC-3 immunostaining (0.14 ± 0.25 vs. 4 ± 1 cells/HPF; RA-PL vs hyperoxia-PL; *P* < 0.05; *N* = 10 animals/group), Fig. 6a and b. Expression of the pro-inflammatory cytokine IL-1β, previously shown to play an important role in BPD [40, 41] was also increased in the hyperoxia-exposed pups (RA-PL vs hyperoxia-PL; *P* < 0.05; *N* = 5 animals/group) Fig. 6c. Interestingly, IT administration of JVS-100 to hyperoxia-exposed pups was associated with a decrease in macrophage infiltration (4 ± 1 vs 2.5 ± 1.7 vs cells/HPF; hyperoxia-PL vs hyperoxia-JVS-100; *P* < 0.05; *N* = 10 animals/group), and a reduction in BAL cell count, Fig. 6a-c. This was accompanied by a decrease in the expression of the pro-inflammatory cytokine IL-1β (hyperoxia-PL vs hyperoxia-JVS-100; *P* < 0.05; *N* = 6 animals/group), Fig. 6d, and an increase in the expression of the anti-inflammatory cytokine IL-10 (hyperoxia-PL vs hyperoxia-JVS-100; *P* < 0.05; *N* = 6 animals/group), Fig. 6e.

## Discussion

Despite advances in neonatal care, BPD continues to be a significant health burden. In the Washington State Medicaid program, chronic respiratory diseases costs were generally higher compared to other chronic illnesses, costing the program in excess of 17 million dollars, with the majority of affected children having BPD or sequelae of prematurity [42, 43]. Additionally, in the United States, the overall cost of treating premature infants with BPD is approximately 2.4 billion dollars [42]. There is no cure and BPD survivors face high hospital readmission rates, recurrent respiratory illnesses, and other disabling long-term sequelae [6]. In this study, we demonstrate a potential therapeutic role for SDF-1 in preterm infants with BPD.

We show that JVS-100, a non-viral gene therapy engineered to express SDF-1 attenuates lung inflammation and improves angiogenesis in an experimental model of BPD. We demonstrate that lung SDF-1 gene expression is decreased in neonatal pups with experimental BPD. In addition, we show that IT administration of JVS-100 was effectively delivered to the neonatal lung as evidenced by increased SDF-1 protein expression in treated pups. In keeping with our hypothesis, IT JVS-100 improved angiogenesis and pulmonary hypertension in our model of BPD. These findings were accompanied by a reduction in markers of lung inflammation. Together, these findings not only demonstrate that SDF-1 is a mediator of lung repair in experimental BPD but importantly suggests a possible role for this chemokine in the repair of the injured preterm lung.

SDF-1 is a potent chemoattractant known to play a crucial role in organ development, injury, and repair. In multiple models of injury, SDF-1 enhances organ regeneration by improving angiogenesis [26]. In the present study, we utilized a rodent model of neonatal hyperoxia as it is characterized by lung microvascular damage and decreased angiogenic signalling, similar to that seen in the lungs of preterm infants with BPD [44–46]. We show that following IT JVS-100 treatment, hyperoxia-exposed pups had a modest improvement in angiogenesis as well as an increase in VEGFR2 expression. Consistent with these findings, we also demonstrate increased tube length and branching in hyperoxia-exposed HPMECS treated with recombinant SDF-1. These findings provide further evidence of SDF-1 pro-angiogenic effects and its potential involvement in modulating lung angiogenesis during development.

Interestingly, IT JVS-100 also reduced lung inflammation in experimental BPD. Lung inflammation is a key factor in the pathogenesis of BPD [47–49]. Rindfleisch and colleagues demonstrated that infants who develop BPD have a significant increase in IL-1β [50]. Similar to these findings, in our experimental model of BPD, neonatal pups exposed to hyperoxia also had an increase in IL-1β which was significantly decreased after administration of JVS-100. This was accompanied by an increase in the anti-inflammatory cytokine IL-10 and a decrease in lung macrophage infiltration. Our findings were surprising as other investigators using adult pro-fibrotic disease models have demonstrated that SDF-1 and its receptors have pro-inflammatory effects [9]. They are however in keeping with those of other investigators who show that SDF-1 controls monocyte recruitment to sites of inflammation and also modulates monocyte differentiation towards more pro-angiogenic and anti-inflammatory functions [51]. In addition, SDF-1 regulates IL-10 secretion in T cells by T-cell receptor (TCR) mediated activation of the mitogen-activated protein kinase cascades (MEK-1/ERK) signalling pathway [52].

Another important finding in our study was a modest improvement in alveolar structure. BPD is characterized by disordered angiogenesis and alveolar simplification. Inhibition of angiogenesis impairs alveolarization in the developing rat lung [53] and in experimental BPD, IT administration of vascular endothelial growth factor promotes angiogenesis and preserves alveolar structure [54] suggesting a link between angiogenesis and alveolarization. Inflammation decreases alveolar septation [55, 56] and transgenic adult mice engineered to express the proinflammatory cytokine IL-1β have lung emphysematous changes [57]. Our current findings of a modest improvement in alveolar structure suggest that SDF-1 may promote alveolar repair by blunting the inflammatory response and augmenting angiogenesis.

One of the most frequent causes of mortality in BPD is pulmonary hypertension [58–61]. In our current study, IT JVS-100 decreased RVSP, vascular remodelling and right ventricular hypertrophy. While the mechanisms by which PH develop in BPD are still being elucidated, inflammation is a significant player. Patients with pulmonary hypertension have increased levels of IL-1β [62] and overexpression of proinflammatory cytokines induces pulmonary hypertension [63]. We speculate that SDF-1 reduction in inflammation and improvement in angiogenesis may be potential mechanisms by which SDF-1 reduces PH in experimental BPD.

Our study has several limitations. Our model of BPD is severe. Most babies are usually exposed to lower oxygen concentrations and therefore further studies evaluating milder forms of this disease will be necessary. In addition, although hyperoxia plays a role in the pathogenesis of BPD, there are several other factors including hypoxia, pre-and postnatal exposure to infection, mechanical ventilation and poor nutrition which contribute to this disease. It is therefore possible that the additive effects of these insults on the preterm lung may potentially alter the efficacy of this therapy. Moreover, there are also important differences between our rat model of BPD and the lung disease evidenced in most preterm infants today. In our present study, rat pups were exposed to hyperoxia from birth to P14, which corresponds to the saccular-alveolar stage of lung development as compared to preterm infants at highest risk for BPD who are mostly in the saccular stage of lung development, with repair and recovery occurring in the alveolar phase. Additionally, JVS-100 is non-viral gene therapy and thus its safety and efficacy in preterm neonates would need to be evaluated. However, JVS-100 use in the myocardium is well studied and has been shown to be safe and effective in patients with ischemic cardiomyopathy [33, 34]. Furthermore, although we demonstrated that early administration of JVS-100 attenuated lung injury, it will be crucial to perform studies evaluating later administration of JVS-100. It is also important to note that further dose finding studies will be crucial as JVS-100 did not return lung structure to normal and it is not known whether higher doses may have deleterious effects. Our study demonstrates an anti-inflammatory and pro-angiogenic role of SDF-1 after neonatal hyperoxia-induced lung injury. But in other studies, SDF-1/CXCR4 signaling potentiates lung inflammation [59, 60]. While further studies will be needed to evaluate the effects on SDF-1 on the different macrophage populations within the lung, we speculate that the effects of SDF-1 and its receptors maybe context dependent as most of the other studies were in adult models and SDF-1 may have different roles during development, homeostasis and repair. Indeed, alterations in SDF-1 monomer-dimer equilibrium in different disease states may modulate SDF-1 receptor interaction, signaling, and cell function leading to varying biological effects [29]. After acute injury, selective activation of SDF-1/CXCR7 signaling pathways promotes liver and lung regeneration, however following chronic injury, SDF-1/CXCR4 signaling pathways dominate, provoking fibrosis. Potentially, in our model, early administration of SDF-1, predominantly activated CXCR7 signaling pathways but further studies evaluating SDF-1 signaling, its effects on lung architecture during varying stages of neonatal hyperoxia-induced lung injury will be necessary. Lastly, although not within the scope of this present study, SDF-1 may also improve lung injury by inducing stem cell homing [17, 64, 65]. Cell based therapies which modulate SDF-1 secretion, have been shown to induce stem cell migration and facilitate organ repair [66]. It is therefore plausible that the findings in our study after IT administration of JVS-100 may be secondary to its effect on stem cell migration and modulation .

## Conclusion

The present study provides evidence of a potential reparative role of SDF-1 in neonatal hyperoxisa-induced lung injury. We show that administration of JVS-100, a naked plasmid over-expressing SDF-1, improves lung structure and angiogenesis in rodents with experimental BPD. Although further investigation will be needed, the present study demonstrates that therapies which modulate SDF-1 expression may be potentially efficacious for the prevention and treatment of BPD.

## Data Availability

Please contact author for data requests.

## Abbreviations

BAL: bronchoalveolar lavage fluid
BPD: bronchopulmonary dysplasia
CXCL12: chemokine ligand 12 aka SDF-1
CXCR4: chemokine receptor 4
CXCR7: chemokine receptor 7
HPF: high power field
HPMEC: human pulmonary microvascular endothelial cell
IT: intra-tracheal
PH: pulmonary hypertension
RA: room air, normoxia
RV/LV + S: weight ratio of the right ventricle to left ventricle and septum
RVSP: right ventricular systolic pressure
SDF-1: stromal derived factor-1
SPC: surfactant protein C
VEGF: vascular endothelial growth factor
α-SMA: anti-α-smooth muscle actin
